# A real-time deep learning method for detection and localization of non-emerged cabbage seedlings

**DOI:** 10.3389/fpls.2026.1984989

**Published:** 2026-10-28

**Authors:** Xin Chen, Kang Han, Teng Liu, Xiaotong Kong, Aniruddha Maity, Peng Peng, Changan Di, Jialin Yu, Maolin Yu

**Affiliations:** 1 School of Mechanical Engineering, Nanjing University of Science and Technology, Nanjing, Jiangsu, China; 2 National Engineering Research Center of Biomaterials, Nanjing Forestry University, Nanjing, China; 3 Shandong Laboratory of Advanced Agricultural Sciences, Peking University Institute of Advanced Agricultural Sciences, Weifang, Shandong, China; 4 Department of Crop, Soil and Environmental Sciences, Auburn University, Auburn, AL, United States

**Keywords:** cabbage (Brassica camperstris L.), deep learning, non-emerged seedling, UAV, video processing

## Abstract

Real-time detection and localization of non-emerged seedlings play a pivotal role in effective vegetable crop management. Traditionally, checking the non-emergence of crop seedlings relies on labor-intensive and time-consuming manual inspections. To address this issue, this paper introduces a precise, rapid, and real-time method for detecting and locating non-emerged seedlings using an unmanned aerial vehicle (UAV). The proposed Enhanced YOLOv8n detector improves upon the base model through a more efficient backbone and a bidirectional feature-fusion neck, achieving a mAP50–95 of 81.4%, an 8% improvement over the base model, while preserving real-time inference capability. Furthermore, integrating the Enhanced YOLOv8n, the YOLO tracking algorithm, and image processing algorithms enabled the development of a robust real-time detection and localization method for non-emerged seedlings. The recognition accuracy for non-emerged cabbage seedlings reached 96.9%. Notably, this method can distinguish the quantity of consecutive non-emerged seedlings, demanding only one-fifth of the manual effort, demonstrating substantial potential for practical applications. In summary, the UAV-deployed real-time detection and localization method exhibits excellent capability in detecting and locating non-emerged cabbage seedlings, thus significantly reducing the manpower needed for crop management.

## 1 Introduction

As one of the most widely cultivated vegetable species globally, Chinese cabbage (Brassica rapa L. ssp. pekinensis) has attracted considerable attention from both agricultural researchers and practicing farmers. Its prominence as a staple crop derives from several advantageous traits: a condensed growth cycle enabling multiple harvests annually, broad climatic adaptability spanning temperate to subtropical zones, and consistently high yields per unit area (Kong et al., 2020; Abou-Hussein, 2012). Furthermore, cabbage is routinely incorporated into rotation systems, where it contributes to disrupting pest and pathogen life cycles while replenishing soil organic matter (Nair and Delate, 2016; Benincasa et al., 2017). Year-round availability helps stabilize regional vegetable supply chains (Singh, 2018). In particular, Chinese cabbage occupies a central role in domestic diets due to its nutritional richness, cost-effectiveness, and culinary versatility, driving the expansion of large-scale commercial cultivation nationwide (Lee et al., 2004).

Optimal plant density is a critical factor in field-scale vegetable production, as it directly influences both land-use efficiency and the spatial conditions necessary for healthy plant development (Manasa et al., 2017). During the early seedling establishment phase, cabbage stands frequently suffer losses attributable to environmental stressors—including erratic weather patterns, soil-borne diseases, and herbivore pressure (Perugachi-Diaz et al., 2021). Timely gap-filling through supplemental transplanting compensates for these losses, restores canopy coverage, suppresses weed colonization, and conserves soil moisture, ultimately contributing to higher seasonal yields (Blanco‐Canqui et al., 2022). Detecting these gaps, however, remains a logistical challenge: conventional field scouting across large plantings demands substantial labor input, and human inspectors inevitably miss small patches of missing seedlings, particularly under time constraints (Su et al., 2023). This detection delay often means the optimal replanting window is lost. Computer vision-based detection systems therefore represent a promising alternative for automating early-stage seedling assessment.

Early efforts in automated agricultural plant detection relied predominantly on classical computer vision methodologies. Researchers have investigated feature-based detection strategies leveraging color spectral signatures (Tang et al., 2016; Hamuda et al., 2016), morphological shape descriptors (Bosilj et al., 2018), multispectral reflectance patterns (Pantazi et al., 2017), and surface texture metrics (Bakhshipour et al., 2017). However, the inherently complex nature of field environments—combined with the morphological similarity between young crop seedlings and co-occurring weed species—has consistently limited the detection and classification accuracy of these handcrafted-feature approaches (Wang et al., 2019a).

Over the past decade, deep learning—a sub-discipline of artificial neural networks—has achieved breakthrough performance across a broad spectrum of applications, ranging from natural language understanding (Vaswani et al., 2017; Devlin et al., 2018) to computer vision tasks (Krizhevsky et al., 2012; Dosovitskiy et al., 2020). The core strength of deep neural architectures lies in their capacity to automatically learn hierarchical feature representations directly from raw data, thereby capturing complex patterns that manual feature engineering typically misses (Pak and Kim, 2017; Peng et al., 2026). This rapid progress has been substantially enabled by continuous hardware innovations, especially the exponential growth in GPU computational throughput, which has rendered the training of large-scale networks increasingly practical (Bao et al., 2017).

A growing body of research has explored the application of deep learning models across diverse agricultural scenarios, yielding consistently encouraging outcomes. Representative use cases include plant disease identification (Saleem et al., 2019, 2020; Duan et al., 2026; Lei et al., 2026), quantitative crop phenotyping (Ubbens and Stavness, 2017; Aich et al., 2018), and automated weed species detection (Jin et al., 2021, 2022a, b, c, 2023a; Zhu et al., 2025; Zu et al., 2025). For example, Jin et al. (2023b) deployed EfficientNet-v2 and ResNet architectures for weed recognition, achieving classification accuracy exceeding 0.992 in controlled evaluation. Their work further demonstrated that coupling the trained network with an autonomous sprayer enabled targeted herbicide application. In a similar vein, Zheng et al. (2018) designed a YOLOv3-based recognition system capable of distinguishing seven vegetable types at 38 frames per second with a mean average precision of 87.79%. Taken together, these studies indicate that data-driven detection approaches substantially outperform traditional methods in agricultural contexts (Kamilaris and Prenafeta-Boldú 2018).

Convolutional neural networks (CNNs) have also been successfully adapted for the specialized task of plant counting in agricultural imagery (Bogomasov and Conrad, 2021). As an early example, Häni et al. (2018) demonstrated the feasibility of shallow AlexNet for enumerating apple (Malus) fruits by classifying cluster images into size categories and aggregating predictions. Wang et al. (2019b) developed MangoYOLO for mango (Mangifera indica L.) fruit counting through a detection-tracking-integration pipeline: individual fruits were detected frame by frame, temporally linked using the Hungarian assignment algorithm, and their trajectories predicted via Kalman filtering to suppress duplicate counts. This approach yielded a double-count error of 9.9% and a missed-count rate of 7.3%, corresponding to a net overcount of only 2.6% — a clear improvement over manual counting for commercial orchard-scale operations. More recently, Lin et al. (2022) integrated YOLOv5 with DeepSort to create a real-time peanut (Arachis hypogaea L.) seedling counter operating on UAV video streams.

Proper maintenance of extensive cabbage fields requires the prompt identification and replanting of bare spots (Manasa et al., 2017). Current approaches in this domain tend to prioritize seedling enumeration within static images but fall short in terms of spatial localization and temporal tracking of missing seedlings, which constrains both their accuracy and practical deployment efficiency.

To address the above issues, two innovations are proposed in this work:

1. Development of an Enhanced YOLOv8n model for swift and precise detection of cabbage seedlings.
2. Implementation of a real-time detection and localization method for non-emerged cabbage seedlings, leveraging the Enhanced YOLOv8n, YOLO tracking algorithm, and image processing algorithms. This method facilitates rapid identification of both the occurrence location and condition of seedling deficiencies, improving the efficiency and accuracy of replenishment operations.

## 2 Materials and methods

### 2.1 Dataset preparation

Field imagery was captured across several cabbage cultivation plots from September through October 2023 at the experimental research station operated by the Peking University Institute of Advanced Agricultural Sciences in Weifang, Shandong Province, China (36˚30′N, 119˚15′E). Seeds were planted during August 2023, a period characterized by mean daily temperatures of approximately 26–27 °C and cumulative rainfall of 25.9 mm. In addition to natural precipitation, supplemental drip irrigation was provided from a nearby reservoir to maintain adequate soil moisture throughout the seedling growth stage.

Aerial documentation of crop canopy conditions was performed using a DJI Phantom 4 RTK SE (CN) Combo unmanned aerial system (Shenzhen DJI Innovative Technology Co. Ltd.). The UAV operated in a nadir-oriented flight pattern at a constant altitude of 3 m above the crop surface. The onboard 20-megapixel imaging sensor recorded a total of 1,188 still photographs (4,000 × 3,000 pixels) and continuous video sequences (3,840 × 2,160 pixels at 30 fps) covering the experimental plots.

Object detection tasks require precise spatial localization of each target instance. To this end, bounding-box annotations were generated for all cabbage seedlings appearing in the collected image set. We utilized Anylabeling (v0.3.3)—an open-source annotation platform (https://github.com/vietanhdev/anylabeling) that incorporates the Segment Anything model (Kirillov et al., 2023) for automated mask-to-box conversion—to produce JSON files containing class labels and bounding-box coordinates. Custom Python scripts were then used to reformat these JSON outputs into the text-file format required by YOLO training pipelines. The final dataset was randomly partitioned into training (832 images), validation (238 images), and testing (118 images) subsets following a 7:2:1 split ratio.

### 2.2 Enhanced YOLOv8n

YOLOv8 stands at the forefront of state-of-the-art (SOTA) object detection models, excelling in both detection speed and accuracy. However, considering the high computational cost associated with deploying devices in practical fields, this study selected YOLOv8n, the smallest network model within the YOLOv8 series with the fewest computational parameters. Despite this, our preliminary tests revealed that YOLOv8n still faces challenges related to lower accuracy (Bai et al., 2023). Additionally, due to the limited computational performance of the deployed devices, achieving real-time detection remains elusive.

To address these issues, YOLOv8n was improved in two aspects, as illustrated in **Figure 1**:

**Figure 1:**
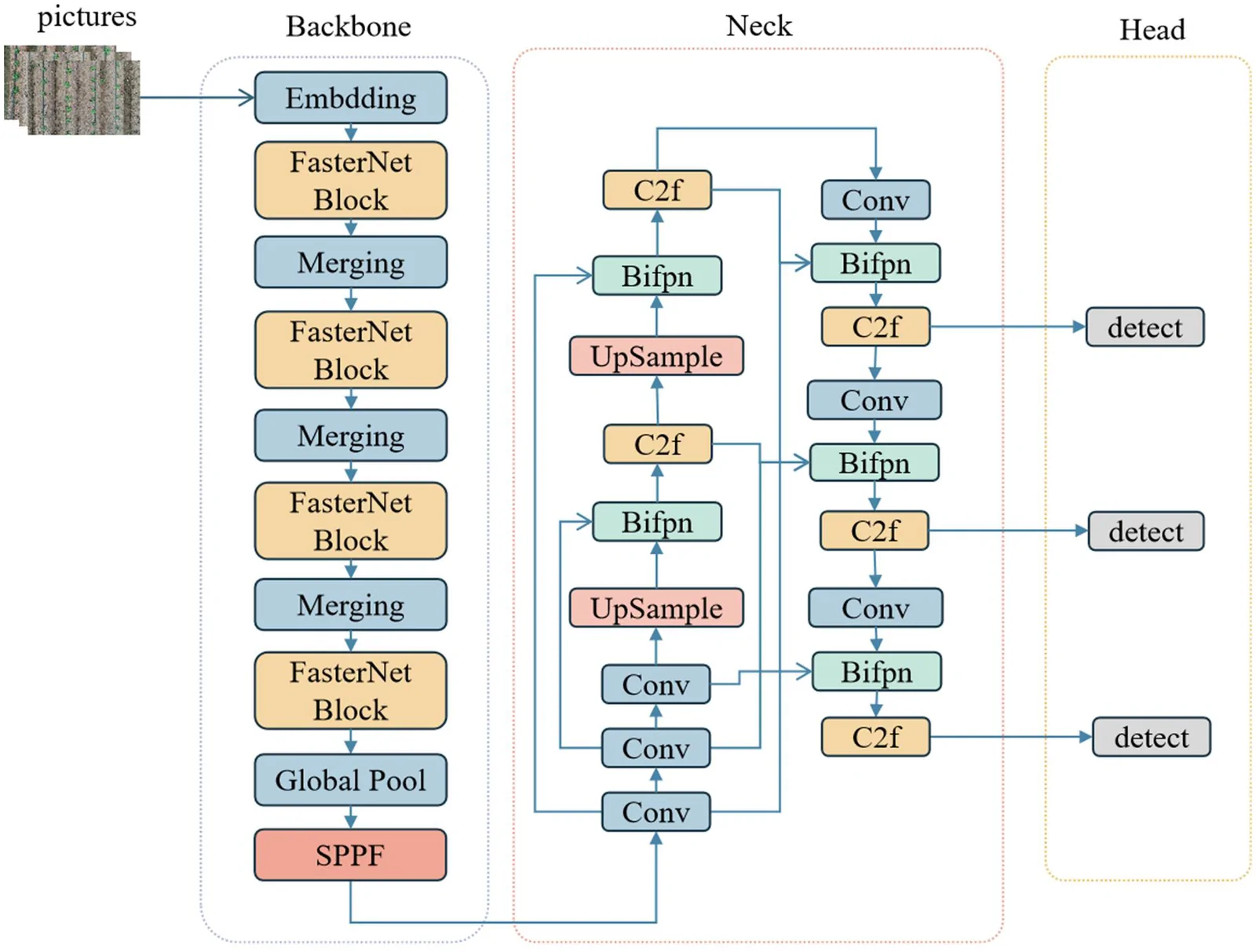
The structure of the Enhanced YOLOv8n. SPPF, Spatial Pyramid Pooling Feature Module; Conv, Convolutional Block.

- The backbone of YOLOv8n was replaced with FasterNet (Chen et al., 2023) for efficient feature extraction.
- The concat operations were substituted with a Bi-directional Feature Pyramid Network (BIFPN) (Tan et al., 2020) to leverage all available feature information.

FasterNet, a cutting-edge neural network architecture, was selected to replace the backbone of YOLOv8n. This architecture is specifically designed to enhance the efficiency and effectiveness of feature extraction in object detection tasks. One of its notable advantages is its ability to expedite the extraction of crucial features from input images, enabling faster and more accurate detection of objects. The structure of FasterNet Block is demonstrated in **Figure 2**.

**Figure 2:**
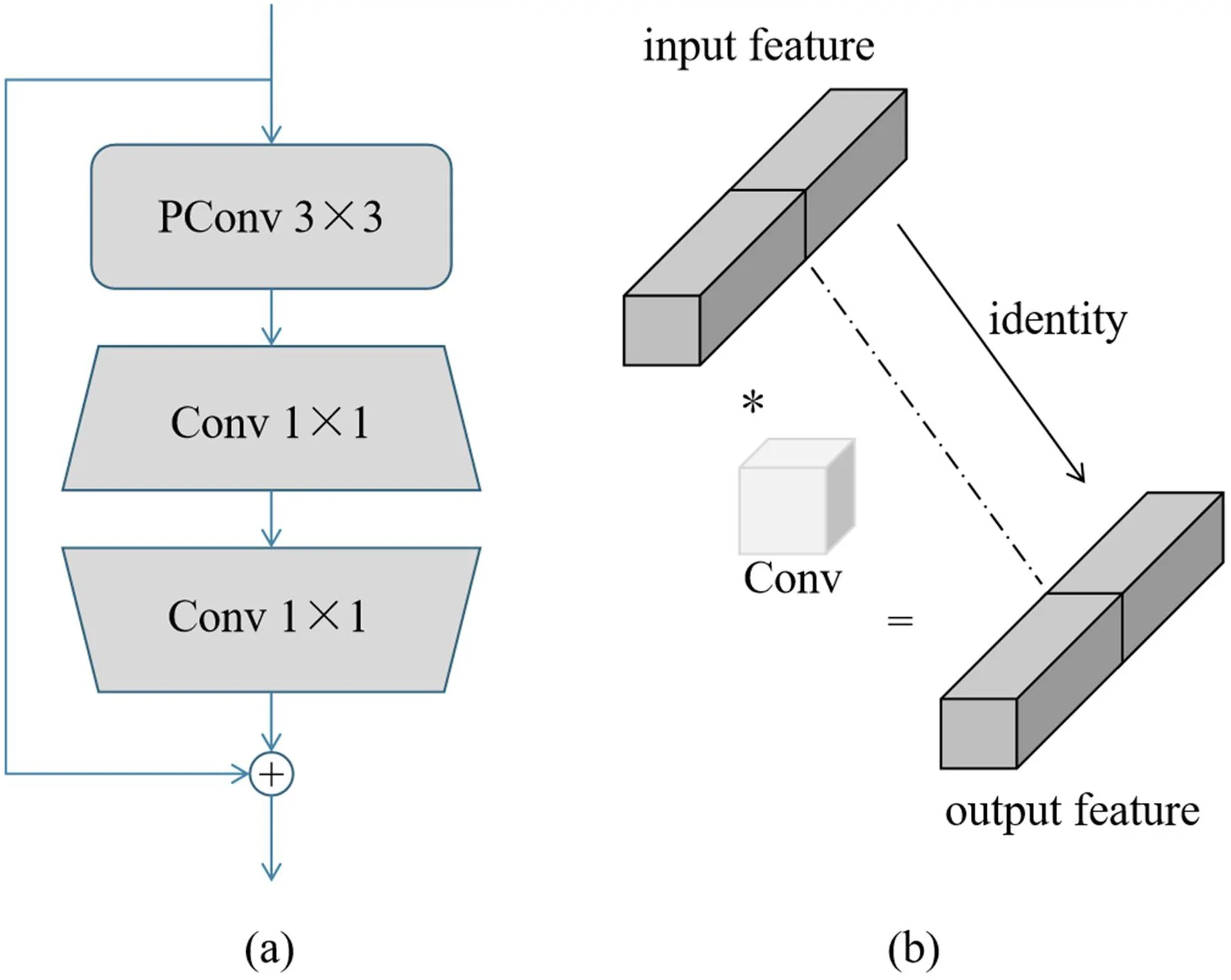
The structure of FasterNet Block. **(a)** Whole structure. **(b)** The structure of PConv in subfigure **(a)**. PConv, Partial Conv; Conv, Convolutional Block.

A key design element of FasterNet is its Partial Convolution (PConv) operator. Unlike conventional convolution, whose frequent memory accesses—especially in depthwise convolution—limit the achievable FLOPS, PConv simultaneously reduces redundant computation and memory access, thereby extracting spatial features more efficiently. By maximizing the realized FLOPS rather than merely minimizing the nominal FLOPs count, this design enables FasterNet to run substantially faster on real hardware (GPU, CPU, and ARM) without sacrificing accuracy.

When choosing the replacement backbone, we prioritized realized inference speed on edge hardware over the nominal FLOPs count. MobileNetV3, ShuffleNetV2, and GhostNet are widely used lightweight backbones, but many of their efficient operations (e.g., depthwise separable convolution) are bottlenecked by frequent memory access rather than by arithmetic, so their low FLOPs do not always translate into proportional latency reductions. FasterNet, in contrast, is explicitly designed to maximize the realized FLOPS by removing redundant computation and memory traffic through PConv, and it exposes a conventional hierarchical feature structure that integrates cleanly with the YOLOv8 neck. These properties—high real-hardware throughput, a favorable FLOPS-to-accuracy ratio, and direct compatibility with the existing neck—made FasterNet the most suitable backbone for our real-time, edge-deployed detector.

Additionally, a BIFPN was adopted to improve the performance of YOLOv8n. The structure of BIFPN is shown in **Figure 3**. By replacing the concat module and adding more bridges between multiple feature layers, BIFPN was integrated into the Neck of YOLOv8n. One notable advantage of BIFPN is its capability to fuse features from different levels with a bi-directional approach, allowing for a more comprehensive integration of multi-scale information. This bidirectional feature fusion mechanism contributes to a richer understanding of contextual information and hierarchical features within the input data, improving object detection accuracy. When integrated into YOLO as a replacement for the concat operation, BIFPN facilitates the amalgamation of features from diverse network levels in a more informed and coherent manner. This strategic feature fusion enables the model to capture intricate details, adapt to varying object scales, and enhance its overall ability to detect objects across different contexts.

**Figure 3:**
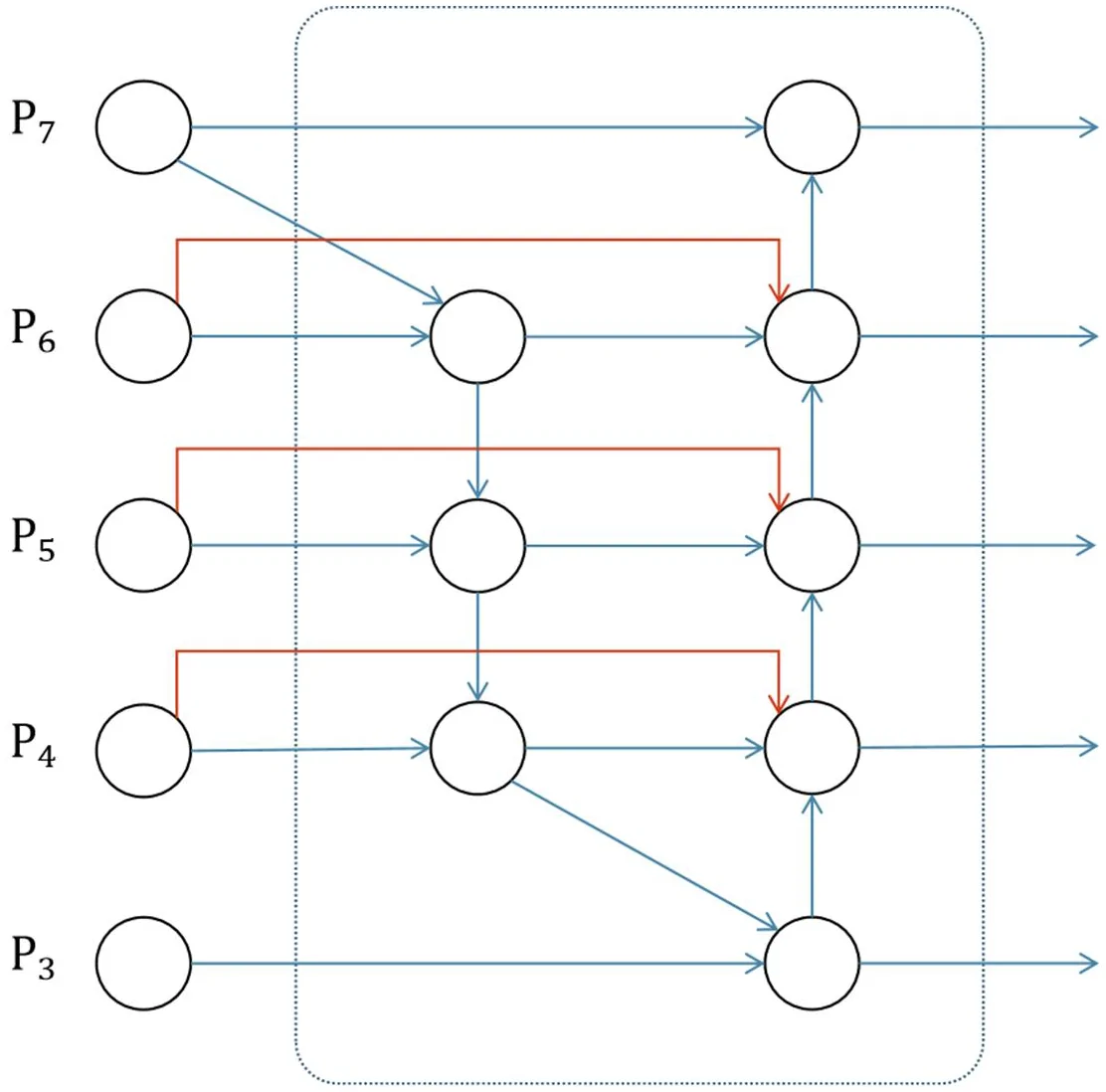
The structure of the Bifpn.

### 2.3 YOLO tracking algorithm

To achieve effective tracking, it is crucial to distinguish the same object in the neighboring frame. This study introduced the concept of “cascade match” in DeepSort (Wojke et al., 2017) into our tracking algorithm to prevent ID switches of the identical instances.

The conventional approach utilizes a Kalman filter algorithm to anticipate the next frame’s potential target position by analyzing the target’s motion information from the preceding frame. Subsequently, Intersection over Union (IoU) matching is applied to compare the predicted position with the detected target position, assessing the area overlap. A successful match (IoU surpassing a threshold) indicates similar positions in both frames, suggesting the possibility of the same target. However, ID switches can still occur. Therefore, a feature vector match was added as the cascade match in our tracking algorithm.

The complete YOLO-based tracking pipeline executes through the following sequential stages: First, the Enhanced YOLOv8n detector localizes all cabbage seedling instances in each video frame while simultaneously extracting deep feature embeddings for every detection. Second, the Kalman filter uses the bounding-box state from the previous frame as initialization to predict the expected location and scale of each tracked object in the current frame. Third, a primary spatial association computes the IoU between predicted and observed boxes; candidate pairs exceeding the IoU threshold advance to the next stage. Fourth, a secondary appearance-based matching computes the cosine similarity between deep feature vectors of temporally adjacent detections, further verifying that spatially proximate detections correspond to the same physical object. This feature-level verification serves as the decisive factor in establishing identity continuity when spatial proximity alone proves ambiguous. Finally, once associations are confirmed, the Kalman filter state is updated, dynamically refining predictions throughout the tracking sequence.

By combining motion-based state prediction, spatial overlap assessment, and appearance feature verification in a multi-stage association framework, the system maintains robust target identity continuity across extended tracking sequences. The overall workflow of the YOLO tracking algorithm is illustrated in **Figure 4**.

**Figure 4:**
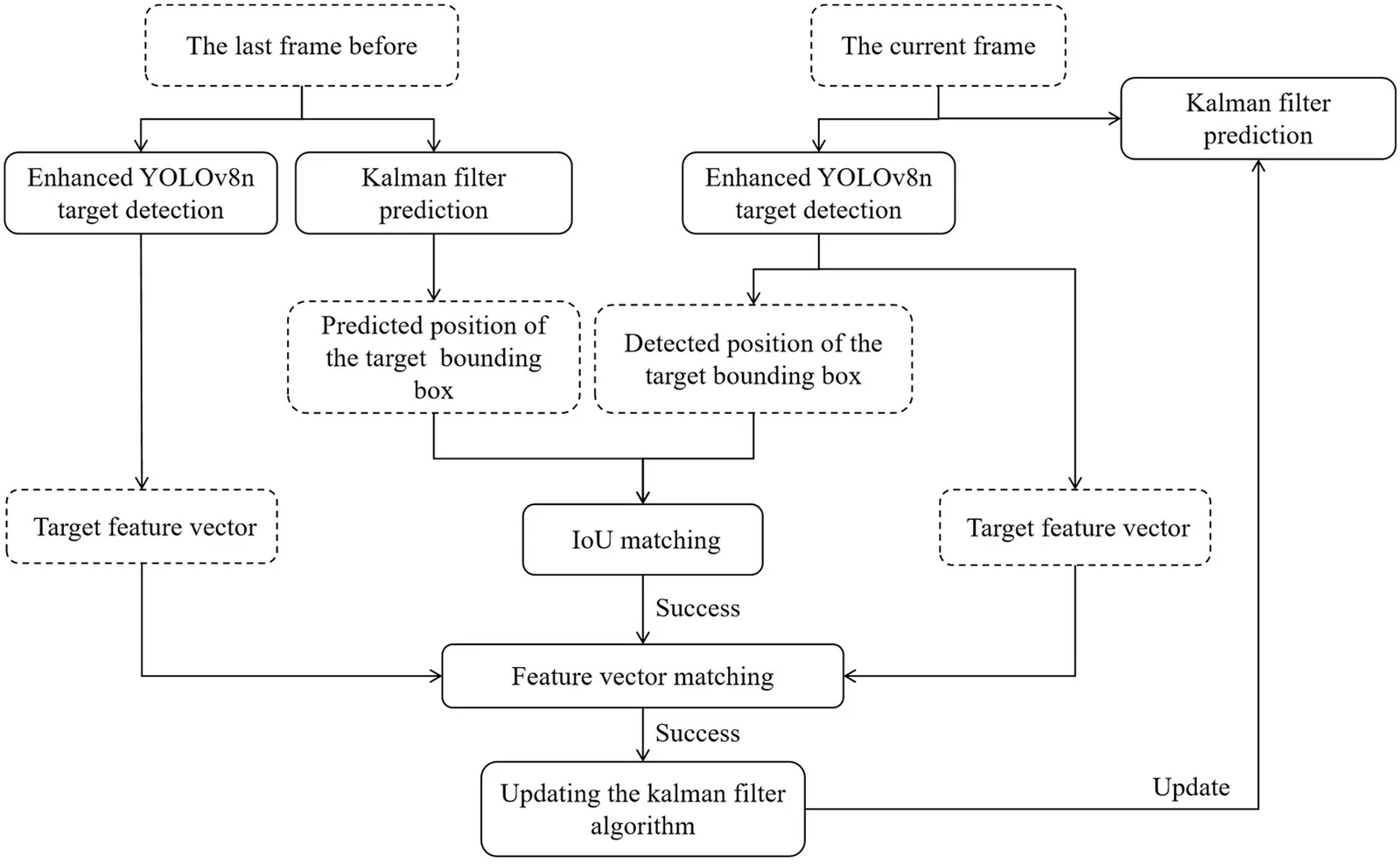
The workflow of the YOLO tracking algorithm. IoU, Intersection over Union.

### 2.4 Real-time detection and localization method for non-emerged seedlings

The real-time detection and localization method for non-emerged seedlings consists of three main components: the Enhanced YOLOv8n, the YOLO tracking algorithm, and the image process algorithm.

For clarity of description, we assume the UAV flies parallel to crop rows in the field. The detection and localization pipeline executes the following sequential image processing steps: (a) Seedling tracking: The combined Enhanced YOLOv8n and tracking algorithm continuously monitors each visible cabbage seedling throughout the video sequence. The midpoint of the bottom edge of each seedling’s bounding box is designated as a tracking reference point. A virtual detection line is established perpendicular to the UAV flight direction. (b) Coordinate system definition: When a reference point’s trajectory first intersects the detection line, that intersection is designated as the origin (point O) of a local coordinate frame. The UAV flight direction defines the positive Y-axis, and the positive X-axis follows from a 90°clockwise rotation. All reference points whose absolute X-coordinate values fall within 500 pixels of the origin are grouped as belonging to the same crop row. (c) Inter-seedling distance computation: The Euclidean distances between consecutive reference points along a row are calculated. The point with the minimum distance from the current reference is identified as the next seedling in that row. When this inter-point distance substantially exceeds a predefined planting-interval threshold (500 pixels in our experimental setup), a gap indicating one or more missing seedlings is flagged at that location. Moreover, if the distance exceeds twice the threshold, two consecutive missing seedlings are inferred, and this logic extends proportionally for larger gaps. (d) Gap visualization: Using the planting-interval threshold and the current reference point as anchors, expected seedling positions within identified gaps are estimated and highlighted with blue rectangular bounding boxes. Frames containing detected gaps are additionally framed with a red border to provide visual alerting. (e) Sequential frame iteration: Steps (b) through (d) are iteratively applied to successive frames until the entire video sequence has been processed. The 500-pixel threshold employed here serves as an illustrative example; in practical deployment, this parameter should be calibrated according to actual planting spacing and the UAV’s flight altitude. The complete procedural flow is depicted in **Figure 5**.

**Figure 5:**
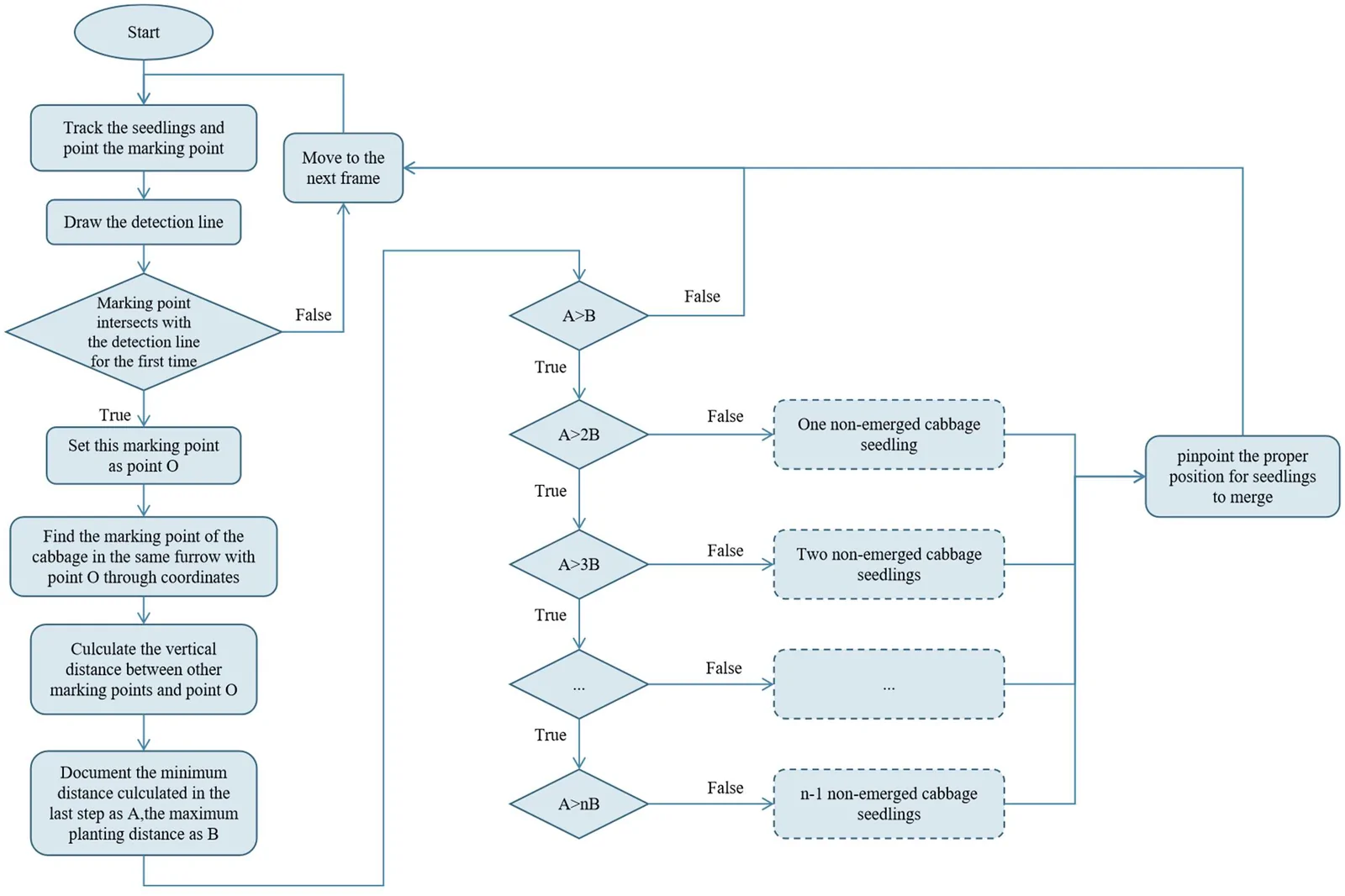
Specific procedure of real-time detection and localization method.

### 2.5 Experimental configuration

The experiments were conducted using consistent computational hardware and software settings to eliminate potential confounding factors. The system configuration for experimentation is listed in **Table 1**. To compare the performance parameters of neural networks under the same training volume, we conducted multiple trials to determine the training volume at which most networks converge. By maintaining consistent training parameters, we compared the performance of all networks. The hyperparameters used for training are as follows: During the 100 training epochs, each batch consisted of 16 samples, with the number of workers set to 2. The Adam optimizer, a widely used adaptive learning rate optimization algorithm in deep learning, was utilized due to its ability to handle sparse gradients and dynamically adjust the learning rate for each parameter. The beta value of the optimizer was set to 0.9 to control the exponential decay rate of the moving average. An initial learning rate of 0.002 and a weight decay of 0.005 were set to prevent overfitting. A validation-based early-stopping strategy was employed, so that training terminated early once the validation loss stopped improving. The learning rate followed a step-decay schedule. No data augmentation (such as mosaic, random flipping, or color jitter) was applied during training. The input resolution for both training and inference was set to 2560 × 1440 pixels.

**Table 1:** The system configuration for experimentation.

Component	Specification
Operating system	Ubuntu 20.04.6 LTS
CPU	Intel(R) Xeon(R) W-2265
GPU	Nvidia GeForce RTX 3080Ti
Video Random Access Memory	12 GB
Code running environment	Python 3.9.18, PyTorch 2.0.1, Torchvision 0.14.1
Computing platform	NVIDIA CUDA 11.7

### 2.6 Evaluation metrics

A binary classification confusion matrix, including true positives (TP), false positives (FP), true negatives (TN), and false negatives (FN), was utilized to present the training and testing outcomes of the convolutional neural networks. To evaluate and compare the performance of these networks, metrics such as precision, recall, mAP50, mAP50-95, and inference time were used, as detailed in (Equations 1–3).

(1) $$precision=\frac{TP}{TP+FP}$$

(2) $$recall=\frac{TP}{TP+FN}$$

(3) $$mAP=AP=\int_{0}^{1}precisiond\left(recall\right)$$

In the equations above, mAP denotes mean Average Precision. Given that our detection task involves a single target class (cabbage seedlings), mAP is mathematically equivalent to the average precision of that individual category. The four confusion matrix components are defined as follows: TP represents the count of samples correctly identified as cabbage seedlings; FP denotes the number of non-seedling regions erroneously classified as cabbage; FN indicates actual cabbage seedlings that were missed by the detector; and TN refers to correctly rejected non-seedling regions. Intersection over Union (IoU) quantifies the spatial overlap ratio between predicted and ground-truth bounding boxes. The mAP50 metric reports average precision at a single IoU threshold of 0.5, whereas mAP50–95 extends this assessment by averaging precision across 10 IoU thresholds ranging from 0.50 to 0.95 in increments of 0.05, thereby providing a more stringent evaluation of localization accuracy.

## 3 Results and discussion

### 3.1 Ablation experiment

Ablation study outcomes are summarized in **Table 2**. As observed, the mAP50 metric exhibits relatively modest variation across configurations, which is expected given that this lower IoU threshold already sits near saturation for the base YOLOv8n model. A more discriminating picture emerges under the mAP50–95 metric: substituting the YOLOv8n backbone with FasterNet alone yielded a 0.8 percentage point gain, lifting mAP50–95 to 74.2%. More notably, this architectural replacement dramatically accelerated inference speed—reducing per-frame latency from 4.6 ms to 2.1 ms—confirming that FasterNet’s design philosophy of maximizing FLOPS throughput effectively translates to real-world speed gains. Introducing the BIFPN module in place of the Concat operation substantially boosted detection quality, with precision, recall, mAP50, and mAP50–95 reaching 98.4%, 96.8%, 98.4%, and 78.8%, respectively; the trade-off manifested as an increased inference time of 5.1 ms, attributable to the added computational overhead of bidirectional multi-scale feature propagation. The final Enhanced YOLOv8n configuration, combining both architectural modifications, achieved a balanced performance profile: 98.2% precision, 96.2% recall, 98.9% mAP50, and 81.4% mAP50-95—representing an 8 percentage point improvement over the unmodified baseline—while maintaining an inference speed of 4.0 ms per frame, marginally faster than the original YOLOv8n. These results confirm that both architectural modifications made complementary and meaningful contributions to overall performance.

**Table 2:** Ablation experiment results for enhanced YOLOv8n.

Ablation configuration	Precision (%)	Recall (%)	mAP50 (%)	mAP50-95 (%)	Inference time (ms)
YOLOv8n	98.6	97.2	98.4	73.4	4.6
Applying FasterNet	99.1	96.9	98.4	74.2	2.1
Applying BIFPN	98.4	96.8	98.4	78.8	5.1
YOLOv8n +FasterNet +BIFPN	98.2	96.2	98.9	81.4	4.0

These results clarify the distinct contribution of each modification. The FasterNet backbone primarily acts on efficiency: by removing redundant computation and memory traffic through its partial-convolution design, it substantially reduces the per-frame latency of the base model while preserving, and slightly improving, detection accuracy. The BIFPN neck, in contrast, is the accuracy driver: its weighted, bidirectional feature fusion propagates information along both the top-down and bottom-up paths, allowing the detector to combine the fine spatial detail of shallow layers with the rich semantic context of deep layers—a property that is especially valuable for detecting small, partially occluded cabbage seedlings in dense field scenes. The main cost of this richer fusion is additional computation, which is why the BIFPN-only configuration is the slowest of the single-modification variants. When the two modifications are combined, they act complementarily rather than redundantly: the efficient FasterNet backbone keeps the overall computational budget low, so that the extra cost introduced by BIFPN is absorbed, and the final model simultaneously reaches the highest mAP50–95 and a per-frame latency below that of the unmodified baseline. In other words, each module addresses a different bottleneck—backbone efficiency and fusion quality—and it is precisely their joint use that produces the balanced accuracy–speed profile of the Enhanced YOLOv8n.

### 3.2 Comparison between the enhanced YOLOv8n and other SOTA models

**Table 3** presents a head-to-head comparison between our Enhanced YOLOv8n and several representative SOTA detectors, including Faster R-CNN (a two-stage approach), the one-stage YOLOv3, YOLOv5s, and YOLOv9, and the real-time transformer-based RT-DETR. A clear trend emerges from these data: single-stage detectors consistently outperform the two-stage Faster R-CNN across all measured metrics. More importantly, the comparison against the recently proposed YOLOv9 and RT-DETR shows that our Enhanced YOLOv8n remains competitive with, and in fact ahead of, these newer architectures: it attains the highest mAP50–95 of 81.4%, surpassing the runner-up YOLOv9 (80.7%) by 0.7 percentage points, YOLOv5s (80.4%) by 1.0 percentage points, and RT-DETR (78.5%) by 2.9 percentage points, while also achieving the best mAP50 (98.9%). The advantage is most pronounced in inference speed: the Enhanced YOLOv8n processes each frame in just 4.0 ms—roughly one-quarter of YOLOv9 (15.4 ms), one-fifth of RT-DETR (18.5 ms), and one-third of YOLOv5s (11.9 ms)—making it particularly well-suited for real-time UAV-based deployment. Taken collectively, these results demonstrate that, even against more recently proposed detectors, the Enhanced YOLOv8n strikes the most effective balance between detection accuracy and computational efficiency for cabbage seedling identification.

**Table 3:** Comparison between the enhanced YOLOv8n and other SOTA models.

Model	Precision (%)	Recall (%)	mAP50 (%)	mAP50-95 (%)	Inference time (ms)
Faster R-CNN	96.9	93.4	94.2	67.6	51.3
YOLOv3	98	94.5	96.6	71.5	16.7
YOLOv5s	98.5	96.1	97.6	80.4	11.9
YOLOv9	98.1	96.2	98.1	80.7	15.4
RT-DETR	97.4	95.6	97.5	78.5	18.5
Enhanced YOLOv8n	98.2	96.2	98.9	81.4	4.0

### 3.3 Cross-validation

To further assess the robustness of the Enhanced YOLOv8n to random partitioning, we performed 5-fold cross-validation. In each fold, the dataset was partitioned into five subsets of equal size; four were used for training and the remaining one for validation, and this process was repeated five times so that each subset served once as the validation set. The per-fold and overall (mean ± standard deviation) performance of the Enhanced YOLOv8n are reported in **Table 4**. The results show that the model’s performance is stable across folds: mAP50–95 ranges from 80.2% to 82.5% around a mean of 81.4%, and the per-frame inference time ranged from 3.8 to 4.3 ms (mean 4.0 ± 0.2ms), confirming that the reported metrics are not an artifact of a particular random split.

**Table 4:** Five-fold cross-validation results of the enhanced YOLOv8n.

Fold	Precision (%)	Recall (%)	mAP50 (%)	mAP50-95 (%)	Inference time (ms)
Fold 1	98.5	96.8	99.2	82.0	3.8
Fold 2	97.6	95.4	98.1	80.5	4.1
Fold 3	98.8	96.5	99.5	81.8	3.9
Fold 4	97.2	95.0	97.8	80.2	4.3
Fold 5	98.9	97.3	99.9	82.5	4.0
Mean ± Std	98.2 ± 0.7	96.2 ± 0.9	98.9 ± 0.8	81.4 ± 0.9	4.0 ± 0.2

### 3.4 Detection and localization performance of the method

We evaluated the proposed detection and localization framework under actual field conditions. A closely related line of work by Cui et al. (2023) employed neural networks in conjunction with UAV imagery to detect missing rice (Oryza sativa L.) seedlings by classifying images into discrete gap categories (e.g., one missing seedling, two missing seedlings, etc.) and subsequently aggregating per-image gap counts across the entire survey area. While conceptually straightforward, this classification-based strategy carries an inherent limitation: when the actual number of consecutive missing seedlings exceeds the maximum gap category represented in the training annotations, the model cannot enumerate the excess, leading to systematic underestimation. Our approach operates on a fundamentally different principle: rather than classifying gap patterns, we first detect every individual seedling present, then infer the number of missing plants by analyzing spatial intervals between adjacent detections along each row. This instance-level strategy proves inherently more robust to variable gap sizes, circumvents the need for exhaustive gap-pattern annotation, and eliminates the risk of systematic undercounting. **Table 4** reports a side-by-side comparison between manual gap inspection and our automated detection and localization method, evaluated across four crop rows, which contained a total of 796 cabbage seedlings (764 emerged and 32 non-emerged).

As shown in **Table 5**, the automated method attained an overall gap identification accuracy of 96.9%, with only a single false-negative case observed across all four crop rows. This one miss was traced back to strong wind gusts during data acquisition, which caused the flexible cabbage leaves to oscillate and temporarily displaced a tracking reference point beyond the detection threshold. Such weather-induced artifacts are infrequent in practice and can be effectively mitigated by scheduling UAV flights during calm meteorological conditions. In addition to accuracy, the proposed method proved markedly more time-efficient than manual scouting, completing the equivalent inspection task in approximately one-fifth of the time required by human operators. Prolonged manual inspection demands sustained visual concentration, which naturally degrades inspector attention and elevates error rates over extended work periods. By contrast, the computer vision pipeline maintains consistent detection quality throughout the entire video sequence regardless of duration. **Figure 6** illustrates three representative scenarios of successful gap detection and localization.

**Table 5:** Detecting and locating non-emerged cabbage seedlings from the manual inspection and the proposed real-time method.

Methods	Number of non-emerged cabbage seedlings	Accuracy (%)	Time (min)
Manual inspection	32	100	20.7
Real-time detection and localization method	31	96.9	4.3

**Figure 6:**
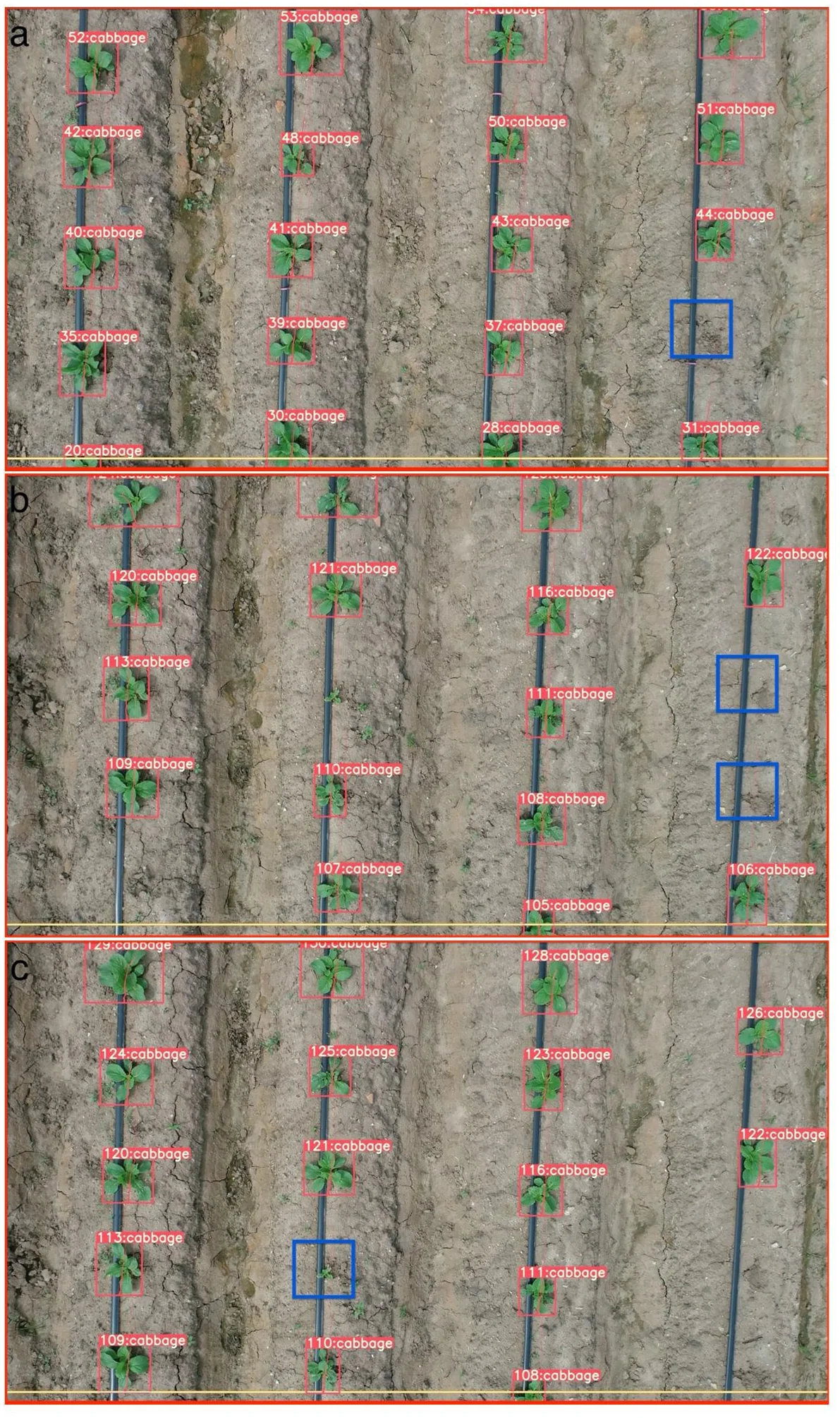
Presentation of three situations of cabbage seedlings detection and localization. **(a)** Detection of one non-emerged cabbage seedling. **(b)** Detection of two non-emerged cabbage seedlings. **(c)** Misidentification of one non- emerged cabbage seedling as weeds.

### 3.5 Limitations

This work has several limitations that should be acknowledged. First, the detection and localization pipeline assumes that crop rows are straight and that the UAV flies parallel to the rows; strongly curved or irregularly arranged rows, or oblique flight paths, would violate these assumptions and could degrade gap localization. Second, the method assumes a uniform planting interval along each row, so fields with variable or irregular spacing would require row-specific parameterization. Third, the 500-pixel threshold used to define the maximum planting distance and row membership is specific to the flight altitude and planting density of this study; deploying the method at different altitudes, with different sensors, or under different planting patterns would require recalibrating this threshold. Fourth, in the present implementation the inference of the Enhanced YOLOv8n was performed on a ground workstation rather than on the UAV’s onboard computer; while the 4.0 ms per-frame latency indicates that real-time on-board processing is feasible, the full real-time pipeline has not yet been deployed on the aircraft itself, which remains an important next step. Future work will address these limitations by integrating the inference pipeline into the UAV platform for true on-board real-time operation, exploring operation from higher vantage points with larger UAVs or fixed-wing platforms, and extending validation to additional crops and seasons.

## 4 Conclusion

Timely and precise identification of missing cabbage seedlings constitutes a critical requirement for efficient crop management in modern vegetable production. Conventional manual scouting is inherently labor-intensive and susceptible to human error, particularly when inspectors must cover extensive field areas under compressed timeframes. In this study, we developed an Enhanced YOLOv8n architecture by replacing the original backbone with the FasterNet module—thereby accelerating feature extraction—and substituting the Concat operation in the feature fusion neck with a BIFPN structure to enable bidirectional multi-scale information propagation. Ablation experiments confirmed that these two modifications collectively elevated mAP50–95 to 81.4%, representing an 8 percentage point increase over the unmodified YOLOv8n baseline, while preserving real-time inference capability at 4.0 ms per frame. Benchmarking against widely used SOTA detectors demonstrated that the Enhanced YOLOv8n ranked favorably across all evaluated metrics. Building upon this improved detector, we further developed a real-time detection and localization framework by integrating the Enhanced YOLOv8n, a Kalman-filter-based tracking pipeline with cascade matching, and row-level spatial analysis algorithms. The integrated system achieved a field-level gap identification accuracy of 96.9%, successfully enumerated consecutive missing seedlings of varying lengths, and required approximately one-fifth of the labor time associated with conventional manual inspection. Deployed on a UAV platform, the proposed framework has the potential to substantially streamline crop monitoring workflows and provide actionable, real-time data for precision agriculture operations at commercial scale.

Furthermore, by integrating the Enhanced YOLOv8n, YOLO tracking algorithm, and the image process algorithms, this work proposed the real-time detection and localization method for non-emerged seedlings. This method achieved a recognition accuracy of 96.9%. Additionally, it can differentiate the quantity of consecutive missing seedlings, requiring only one-fifth of the manual effort, demonstrating substantial potential for practical application. In practical scenarios, the real-time detection and localization method for non-emerged seedlings can quickly locate non-emerged seedlings, assisting subsequent replanting. The implementation of this method has the potential to significantly enhance the efficiency of crop management, offering effective support for large-scale agricultural production.

## Data Availability

The raw data supporting the conclusions of this article will be made available by the authors, without undue reservation.
